# Dataset prepared for characterization of three South African manganese ores before or after preheating in laboratory-scale rotary kiln

**DOI:** 10.1016/j.dib.2022.108566

**Published:** 2022-08-31

**Authors:** M.S. Moholwa, J.D. Steenkamp, H.L. Rutto

**Affiliations:** a MINTEK, 200 Malibongwe Road, Randburg, 2125, South Africa; b Vaal University of Technology, Andries Potgieter Blvd, Vanderbijlpark, 1900, South Africa; c University of the Witwatersrand, 1 Jan Smuts Avenue, Johannesburg, 2000, South Africa

**Keywords:** Decrepitation, Manganese ores, Rotary kiln, Temperature, Rotational speed

## Abstract

Manganese ores are the major raw materials utilized in the production of manganese ferroalloys. A common problem in the production of manganese ferroalloys is the lack of knowledge regarding mineralogical and metallurgical properties of manganese ores. Decrepitation, which is described as the breakage or disintegration of the ore particles upon heating, is an important quality parameter of these ores. The decrepitation Index (DI), which is the parameter used to describe the extent of decrepitation, is described as the ratio of mass of particles <6 mm after pre-heating to the total mass of the sample. The purpose of this paper is to describe all the raw data produced during the course of an investigation into the effect of temperature, rotational speed, and particle size on the decrepitation of three types of manganese ores sourced from South Africa. Furthermore, the relevance of the raw data to scientific community as well as industry is explained. The data set will include sub-sets of data i.e. the characterization of the as-received samples, the decrepitation test results, and the characterization of post experiment samples.

**Specifications Table**

**Table:** 

Subject	Production of High Carbon Ferromanganese alloys
Specific subject area	Decrepitation of SA manganese ores
Type of data	Tables, graphs, SEM micrographs
How data was acquired	Screening of samples into different size fractions Representative sampling to produce sub-samples Determination of physical properties: porosity, moisture content, loss on ignition (LOI), and bulk density Determination of bulk chemical properties: inductively coupled plasma - optical emission spectrometry (ICP-OES) Determination of phase chemical properties: Quantitative X-ray diffraction (QXRD) Determination of decrepitation index (DI), the formula was taken from the ISO standard 8371 used to determine DI of iron ores.
Data format	Raw, analyzed
Parameters for data collection	For three size fractions of three different manganese ores: • Physical properties of natural ores • Bulk chemical properties of natural ores • Bulk phase chemical properties of natural ores • DI for ores heat treated in rotary kiln at different temperatures or rotational speeds
Description of data collection	Manganese ore of specific size range [+6-20, +20-40 or +40-75 mm] were fed into a kiln at specific temperature [600, 800 or 1000°C] and a specific rotational speed [3, 6 or 12 rpm] and maintained there for 30 minutes. After cooling, the ore was screened to determine the amount of < 6 mm particles.
Data Source location	Mintek Johannesburg South Africa 26°05′19″S 27°58′39″E
Data accessibility	Repository name: Mendely Data Data identification number: 10.17632/g6pxkrmrmn.1 Direct URL to data: Decrepitation of Mn ore dataset - Mendeley Data

## Value of the Data

• The data will be useful when a production facility with pre-heater is constructed/ a pre-heater is retro-fitted in an existing facility. The production engineers/scientist will use the data to select ores or ore blends if necessary and kiln process parameters that will ensure the most possible optimum operation of the (SAF) Sub-merged Arc Furnace. Proper selection of ores/ ore blends will also help ensure safety of plant workers.
• Utlilizing these data will help the management of the facility ensure the process runs smoothly with minimum unscheduled shutdowns, this will in turn lower production costs, maximize profits, and improve the economy of the country.
• The data can also be used to further understand the behavior of minerals found in manganese ores when exposed to heat, this can give insights to scientist (mineralogist) who intends to design experiments to study the behavior of different minerals in manganese ores.

## 1 Data Description

Table 1 summarises the porosity, moisture content, and LOI expressed in percentage (measured in triplicate) of natural Ore A, Ore B, and Ore C as well as their bulk density at +6-20mm, and DI at 800°C and 6 rpm.

**Table 1:** Physical properties of Ore A to Ore C.

Ore type	Porosity [%]	Moisture [wt%]	LOI [wt%]	Bulk density [g/cm^3^]	DI [%]
Ore A	0.75	0.83	6.01	1.60	38.36
Std dev	0.01	0.05	0.19	0.02	
Ore B	0.71	0.44	3.49	1.69	20.31
Std dev	0.02	0.01	0.08	0.04	
Ore C	0.74	0.62	3.61	1.78	16.80
Std dev	0.03	0.01	0.11	0.07	

Table 2 summarises the calculated average (of three measurements) and standard deviations of the bulk chemical compositions of the natural Ore A, Ore B, and Ore C as determined by ICP-OES for three representative samples per ore. When analyzing manganese ores it is conventional to present Mn and Fe in elemental form and other components in oxide form due to the complex nature of the Mn and Fe bearing minerals in the ores. It also explains why the total compositions of the ores are significantly less than 100.00%.

**Table 2:** Calculated average and standard deviations (Std Dev) of the bulk chemical compositions of Ore A, Ore B, and Ore C as determined by ICP-OES. Values presented in mass percentage (wt%).

Ore type		Mn	Fe	Al_2_O_3_	CaO	MgO	SiO_2_	Total
Ore A	Average	29.24	5.52	0.25	21.51	3.22	4.84	64.58
	Std Dev	0.23	0.05	0.04	0.15	0.03	0.05	
Ore B	Average	38.87	5.16	0.21	15.42	2.01	4.71	66.38
	Std Dev	0.11	0.04	0.01	0.26	0.01	0.04	
Ore C	Average	35.17	4.57	0.21	16.52	2.99	6.01	65.47
	Std Dev	0.07	0.02	0.01	0.19	0.01	0.04	

Table 3 presents the bulk phase chemical compositions of the nartural Ore A, Ore B, and Ore C as determined by QXRD. Values marked with an asterisk (*) indicate that the absence of a specific mineral in that specific ore.

**Table 3:** Minerals present in natural Ore A, Ore B, and Ore C and their quantities in mass percentage (wt%) as determined by QXRD. The ideal chemical composition of minerals present in ore samples are presented in Table 9.

Mineral	Ore A	Ore B	Ore C
Hematite	4.50	5.20	*
Jacobsite	*	*	6.50
Bixbyite	5.40	5.50	*
Braunite	30.20	40.00	29.80
Hausmannite	6.00	12.30	21.30
Kutnohorite	23.20	10.00	14.40
Calcite	20.00	24.60	24.60
Dolomite	9.80	<1	*
Clinopyroxene	*	*	2.70
Jianshuiite	*	<1	*

**Fig. 1:**
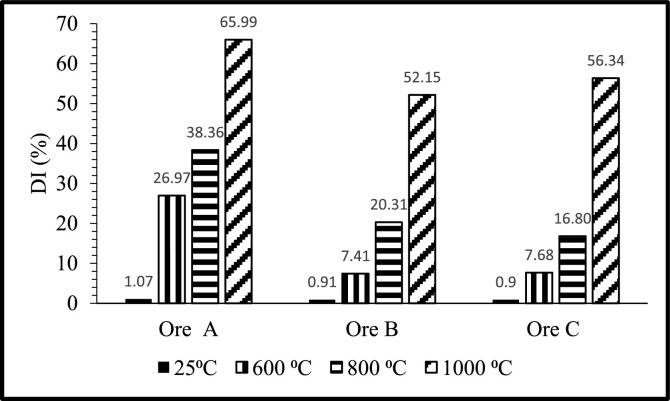
DI's of Ore A, Ore B, and Ore C at different temperatures and with the rotational speed fixed at 6 rpm for the +6-20mm size fraction.

**Fig. 2:**
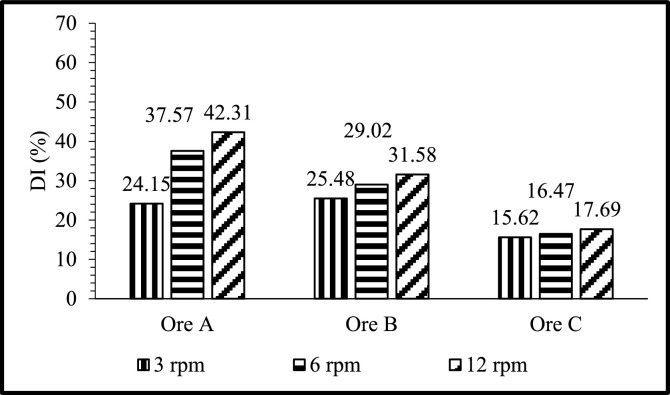
DI's of Ore A, Ore B, and Ore C at different rotational speeds and with the temperature fixed at 800°C for the +6-20 mm size fraction.

**Fig. 3:**
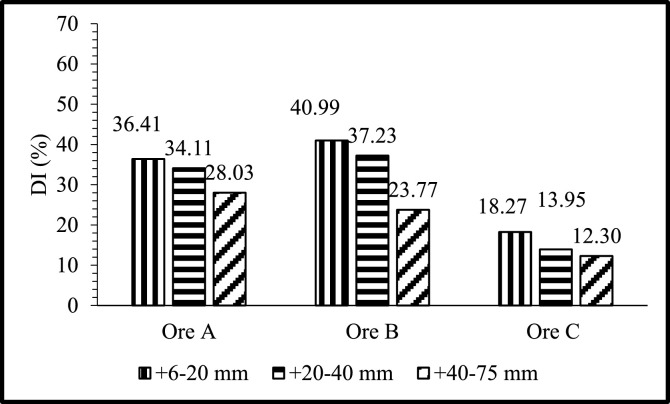
DI's of Ore A, Ore B, and Ore C at different size fractions with temperature fixed at 800°C and rotational speed at 6 rpm.

Fig. 1, Fig. 2, Fig. 3 presents the DI's of Ore A, Ore B, and Ore C at different temperatures (600, 800 and 1 000°C) rotational speeds (3, 6 and 12 rpm) or size ranges (+6-20, +20-40 and +40-75 mm). The DI's determined at room temperature were included as baseline.

To determine the bulk chemical and bulk phase chemical compositions of the samples after heating, each sample was sieved to prepare sub-samples containing the <6 mm and >6 mm size fractions. Table 4 to Table 6**.** present the bulk chemical compositions of the <6 mm and >6 mm size fractions determined by ICP-OES on three sub-subsamples per sub-sample.

**Table 4:** Calculated average and standard deviations (Std Dev) of the bulk chemical compositions, determined by ICP-OES, of the <6 mm and >6 mm sub-samples of Ore A. The samples were heated for 30 minutes at 600, 800, or 1000°C in the rotary kiln with the rotational speed fixed at 6 rpm and the input material being the +6-20mm size fraction. Values presented in mass percentage (wt%).

Ore A						
Sample	Mn	Fe	Al2O3	CaO	MgO	SiO2
	%	%	%	%	%	%
<6 mm						
600°C	27.52	6.67	0.21	20.08	3.92	6.32
800°C	27.67	6.62	0.21	20.01	3.38	7.16
1 000°C	30.09	9.28	0.23	21.32	4.20	6.82
>6 mm						
600°C	31.23	7.91	0.26	22.73	4.13	5.64
800°C	36.24	8.24	0.29	24.95	4.25	6.67
1000°C	37.85	8.87	0.28	22.29	4.09	7.61

Only the >6 mm sub-samples were characterized by QXRD to determine their bulk chemical compositions. The analysis was done for all three ores and the section marked with * show that a specific mineral in a specific ore was not detected.

The data under the “physical properties” tab in the spreadsheet on Mendeley data is similar to the data displayed in Table 1. The only difference is that the online data shows data for individual analysis and averages while the data in Table 1 shows only averages of individual analysis. The same applies to the data in the “ICP-OES” tab, the data is similar to the data shown in Tables 4, 5 and 6. The data sets in the tabs “QXRD” and “ DI charts” are the duplicates of the already shown in the manuscripts, (Table 7).

**Table 5:** Calculated average and standard deviations (Std Dev) of the bulk chemical compositions, determined by ICP-OES, of the <6 mm and >6 mm sub-samples of Ore B. The samples were heated for 30 minutes at 600, 800, or 1000°C in the rotary kiln with the rotational speed fixed at 6 rpm and the input material being the +6-20mm size fraction. Values presented in mass percentage (wt%).

Ore B						
Sample	Mn	Fe	Al2O3	CaO	MgO	SiO2
	%	%	%	%	%	%
<6 mm						
600°C	34.82	5.58	0.16	14.89	3.45	6.13
800°C	38.86	4.47	0.19	15.64	2.18	5.39
1 000°C	37.72	6.63	0.24	18.65	3.35	5.79
>6 mm						
600°C	38.32	5.87	0.22	17.78	3.04	5.50
800°C	41.34	6.93	0.24	18.46	3.61	6.21
1 000°C	43.16	6.71	0.24	17.47	4.71	5.44

**Table 6:** Calculated average and standard deviations (Std Dev) of the bulk chemical compositions, determined by ICP-OES, of the <6 mm and >6 mm sub-samples of Ore C. The samples were heated for 30 minutes at 600, 800, or 1000°C in the rotary kiln with the rotational speed fixed at 6 rpm and the input material being the +6-20mm size fraction. Values presented in mass percentage (wt%).

Ore C						
Sample	Mn	Fe	Al2O3	CaO	MgO	SiO2
	%	%	%	%	%	%
<6 mm						
600°C	37.51	5.51	0.22	16.71	2.58	5.19
800°C	39.52	5.23	0.21	16.14	2.35	4.64
1 000°C	41.37	5.57	0.23	16.48	2.63	5.57
>6 mm						
600°C	41.63	5.35	0.24	16.69	2.47	5.15
800°C	43.88	5.48	0.28	19.21	2.52	5.78
1 000°C	45.40	5.70	0.28	18.31	2.49	5.88

**Table 7:** Minerals present in heat-treated Ore A, Ore B, and Ore C and their quantities in mass percentage (wt%) as determined by QXRD. The +6-20 mm size fraction was heat treated at 600, 800, or 1000°C at 6 rpm for 30 minutes in the rotary kiln. Only the QXRD results for the >6 mm sub-samples were presented here. The ideal chemical compositions of minerals present in ore samples are presented in Table 8.

	Ore A			Ore B			Ore C		
Temperature [°C]	600	800	1000	600	800	1000	600	800	1000
**Mineral**									
Hematite	1.5	3.2	11.0	4.4	4.8	7.7	2.7	7.4	4.3
Magnetite	*	2.6	1.4	*	1.5	5.7	*	1.6	10.1
Bixbyite	*	2.1	*	1.5	2.8	*	<1	<1	*
Braunite	24.7	25.7	6.6	39.1	35.1	12.1	26.0	30.4	26.8
Hausmannite	7.4	24.9	38.2	0.8	23.9	35.7	19.3	30.1	42.5
Kutnohorite	20.0	*	*	30.7	*	*	10.0	*	*
Marokite	*	*	22.5	*	*	24.1	*	*	0.4
Calcite	29.3	41.4	1.5	14.7	32.0	2.3	37.8	29.9	11.4
Dolomite	10.4	*		*	*	*	5.1	*	*
Clinopyroxene	*	*		8.8	*	6.7	4.0	*	*
Brownmillerite	*	*	18.9	*	*	12.4	*	*	*
Manganosite	*	*	*	*	*	*	*	*	4.5

⁎ =Not detected.

**Table 8:** Ideal chemical composition of minerals present in the ore samples as identified by QXRD.

Mineral	Ideal chemical composition
Bixbyite	(MnFe)_2_O_3_
Braunite	Mn^2+^Mn^3+^_6_SiO_12_
Brownmillerite	Ca_2_(Al, Fe)_2_O_5_
Calcite	CaCO_3_
Clinopyroxene	(NaCa)(Mg.Fe.Al)(Al.Si)_2_O_6_
Dolomite	CaMg(CO_3_)_2_
Hausmanniite	Mn_2_O_4_
Hematite	Fe_2_O_3_
Jacobsite	(Fe^2+^Mn^3+^)_2_O_4_
Jianahuite	(Mg.Mn^2+^)Mn^4+^_3_O_7_.3H_2_O
Kutnohorite	CaMn(CO_3_)
Manganite	Fe_3_O_4_
Manganosite	MnO
Marokite	CaMn_2_O_4_

## 2 Methods

This investigation utilized three South African manganese ores form the Kalahari Manganese Field (KMF) named Ore A, Ore B, and Ore C on request of the supplier. In the order of 200 kg samples of each type of ore was sourced for the investigation. The samples were screened to obtain +6-20 mm, +20-40 mm, and +40-75 mm size fractions using Endecotts screens. Each size fraction was coned and quartered to produce 10 kg samples representative of the size fraction in question. The sample is arranged in a cone shape on top of a plastic laid on the floor, a shovel is used to divide the sample into quarters. The 10 kg samples were subsequently split into 1 kg representative samples using a rotary splitter. The 1 kg samples were utilized in the decrepitation tests or for further characterization. The sample were taken for the following characterization techniques; Quantitative X-Ray Diffraction (QXRD), ICP-OES and SEM-EDS.

The samples were taken to determine moisture content, bulk density, porosity and loss on ignition (LOI). The moisture content was determined using the ASTM D2216-19 standard method, 1 kg of the ore sample was weighed and put in an oven to dry overnight at 105°C [1]. After drying and cooling the sample was weighed again to determine the change in mass which was then calculated to be the moisture content of that specific ore. The loss on ignition (LOI) was determined using the standardized method from the ASTM D7348 standard [3]. 100 g of the sample was weighed, the sample was then placed in a muffle furnace and heated to 950°C in an oxygen atmosphere. The sample was the cooled and weighed to determine the change in mass (mass lost due to ignition). The porosity was determined using ASTM D4872 method for He-pycnometry test [2]. A sample of particles ranging between 6 and 20 mm was weighed and added to the sample cup. The sample was then placed into a pycnometer chamber. Helium was added to the system and the system was left to reach equilibrium pressures. The system uses the equilibrium conditions to determine the porosity of the sample. A total of 3 samples were analysed for each ore and the average was calculated. The bulk density was determined using the modified volumetric method. The method involves filling a hopper of a known mass (with a volume of 4 293 cm^3^) with particles of Mn ore and weighing it. The mass of the empty hopper was subtracted from the mass of the hopper and ore to get the mass of the ore. The method was repeated three times to ensure accuracy. The bulk density was calculated according to Eq. (1).

(1) $$\rho =\frac{M}{V}$$

Where,

ρ = bulk density [g/cm^3^]

M = the mass of the particles [g]

V = the volume of the hopper [cm^3^]

The bulk phase chemical analysis was determined by quantitative X-ray diffraction method (QXRD. The method provided an ability to quantify and characterize different crystalline phases as well as amorphous phases that may be present. A Bruker D8 diffractometer with an acceleration voltage of 35 kV and cobalt tube with Fe-low beta filter was used with 2Ɵ angle ranging from 2 to 80 degrees and step size of 0.02°2Ɵ. The samples were then taken for quantitative analysis, the D-500 diffractometer with Cu *K* ά radiation and graphite monochromators was used. The refined parameters included scale factors, back-ground coefficients, peak width and profile parameters and cell dimensions. Values of occupancy factors were set to reflect the ideal stoichiometric compositions.

For the decrepitation tests which used a modified method from [4], the laboratory-scale rotary kiln was switched on with a temperature set point of either 600, 800 or 1000°C, and a rotational speed of either 3, 6 or 12 rpm. Once the kiln was at temperature, 1 kg of ore of either the +6-20 mm, +20-40 mm or +40-75 mm size fraction was fed into the kiln and maintained there for 30 minutes. After 30 minutes the sample was allowed to cool and screened for particles <6 mm. The DI was calculated according to Eq. (2).

(2) DI(<6mm)=(M1/M2)*100

Where:

• DI = Decrepitation index
• M1 = Mass of particles < 6 mm in g
• M2 = Total mass of the sample in g

The experimental plan is tabulated in Table 9. In one set of experiments, the temperatures were varied and the rotational speed and size fraction maintained at 6 rpm and +6-20 mm respectively. For the next set of experiments, the rotational speeds were varied and the temperature and size fraction maintained at 800°C and +6-20 mm respectively. For the final set of experiments, the size fractions were varied and the temperature and rotational speed maintained at 800°C and 6 rpm respectively.

**Table 9:** Experimental plan.

Temperature[°C]	Rotational speed[rpm]	Size range[mm]
600	3	+6-20*
800*	6*	+20-40
1000	12	+40-75

Parameters marked with * are kept constant when investigating the effect of others.

## CRediT Author Statement

Credit is given to the following individuals:

• Dr Joalet Steenkamp for providing conceptualization, supervision, funding acquisition, resourcines and writing-review and editing
• Professor Hillary Limo Rutto for providing conceptualization, supervision and writing-review and editing

## Declaration of Competing Interest

The authors declare that they have no known competing financial interests or personal relationships that could have appeared to influence the work reported in this paper.

## Data Availability

Decrepitation of Mn ore dataset (Original data) (Mendeley Data).
